# Endogenous RNA virus–derived elements exhibit functional divergence in modulating development and virus susceptibility in *Bombyx mori*

**DOI:** 10.1080/21505594.2026.2732370

**Published:** 2026-09-09

**Authors:** Ma-Cheng Zhang, Yi-Lan Li, Shuo Yang, Peng Wang, Yao Xu, He-Ying Qian

**Affiliations:** Jiangsu Key Laboratory of Sericultural and Animal Biotechnology, School of Biotechnology, Jiangsu University of Science and Technology, Zhenjiang, China

**Keywords:** Bombyx mori, Non-retroviral endogenous viral elements, larval development, antiviral immunity, Nanoparticle-mediated RNAi

## Abstract

Non-retroviral endogenous viral elements (nrEVEs) are increasingly recognized as potential regulators of host biology, but their functional roles in Lepidoptera remain largely unexplored. Here, we identified seven nrEVEs derived from four RNA virus families in *Bombyx mori*. All seven nrEVEs were transcriptionally active and exhibited distinct developmental and tissue-specific expression patterns. Their small-RNA profiling revealed that these elements could generate small interfering RNA (siRNA)-like RNAs, primary PIWI-interacting RNA (piRNA)-like RNAs, or ping-pong-associated piRNAs. Functional analyses of the *Totiviridae-*derived BmToEVE and the *Chuviridae*-derived BmChuEVE3 revealed markedly divergent physiological roles. Depletion of either nrEVE delayed larval development and reduced body weight, while differentially affecting reproductive and cocoon traits. Yeast two-hybrid screening and transcriptomic analyses further implicated both nrEVEs with cuticle-associated and reproductive processes. Importantly, BmChuEVE3 knockdown broadly suppressed metabolic and immune responses, accompanied by increased *B.mori nucleopolyhedrovirus* (BmNPV) and *cypovirus* (BmCPV). By contrast, BmToEVE silencing markedly reduced BmNPV accumulation and enhanced host defense-related transcriptional responses. Its effect on BmCPV was comparatively modest and emerged only at the later infection stage. Overall, our findings reveal functional diversification of silkworm nrEVEs and highlight the contribution of virus-derived sequences to developmental regulation and antiviral physiology in a lepidopteran host.

## Introduction

The silkworm *Bombyx mori* is an important lepidopteran model and the foundation of sericulture [1,2], with broad applications in genetics, developmental biology, and biotechnology [3]. Extensive genomic and breeding studies have revealed the genetic basis of many economically important traits [4–7]. Recent high-resolution pan-genome resources have further expanded opportunities to explore genomic diversity and trait-associated variation [8,9]. Despite these advances, viral diseases remain a major constraint on silkworm production [10–12]. *B. mori* is highly susceptible to pathogens such as *B. mori nucleopolyhedrovirus* (BmNPV) and *cypovirus* (BmCPV), which cause substantial losses in sericulture [13,14]. While silkworms employ multilayered antiviral defenses, including RNA interference (RNAi) and NF-κB signaling [15–17], the full landscape of their antiviral regulation remains incompletely understood.

Non-retroviral endogenous viral elements (nrEVEs), which are viral genomic fragments integrated into host genomes, have emerged as potential players in host antiviral immunity and other biological processes [18–20]. Comparative genomic studies have revealed abundant and diverse nrEVEs across arthropods, particularly those derived from RNA viruses [21–23]. Many nrEVEs are considered remnants of ancient viral infections, but some remain transcriptionally active and generate small RNAs (sRNAs) [24–26]. These observations have raised the possibility that viral integrations can be retained and repurposed by their hosts [19,27]. To date, the best-characterized functions of EVEs involve antiviral defense [28–30]. For example, endogenous retrovirus (ERV)-encoded envelope (Env) proteins can block viral entry through receptor competition [31], whereas Gag-derived proteins disrupt viral capsid assembly [32,33]. In parallel, EVE-derived PIWI-interacting RNAs (piRNAs) have been shown to silence viral transcripts in vertebrates [34]]and to reduce cognate viral loads in mosquitoes [35] and other arthropods [24,25]. However, antiviral defense likely represents only one outcome of viral domestication. Several EVEs have been repurposed for developmental innovation. A densovirus-derived nrEVE regulates crowding-induced wing polyphenism in aphids [36], and a recently characterized *Nilaparvata lugens* ToEVE was shown to be translated into a functional protein that enhances fecundity [37]. Thus, nrEVEs can potentially function as both sources of antiviral small RNAs and components of host physiological regulation.

Whether such functions are widespread in Lepidoptera remains unresolved. In *Chilo suppressalis*, multiple nrEVEs are transcriptionally active and give rise to piRNA-like small RNAs, although their biological functions have not been experimentally established [38]. More recently, comparative genomic analysis of four *Spodoptera* species identified diverse nrEVEs, predominantly derived from negative-sense RNA viruses [39]. Only a subset of these elements produced detectable piRNAs, and fewer displayed sequence complementarity indicative of a potential antiviral role [39]. These findings establish nrEVEs as a component of lepidopteran genomes, but direct evidence for their biological functions remains limited. In particular, it is unclear whether nrEVEs have functionally diversified in host development and reproduction. Moreover, it is unknown whether nrEVEs modulate antiviral responses beyond sequence-specific restriction of cognate viruses. These questions are especially relevant in *B. mori*. Its extensive genomic, transcriptomic, and small-RNA resources provide an opportunity to examine nrEVE function at both genomic and organismal levels.

Here, we systematically characterized nrEVEs in *B. mori* (BmnrEVEs) and examined whether these elements contribute to host physiology and antiviral responses. We identified seven transcriptionally active nrEVEs derived from four RNA virus families. Small-RNA profiling revealed that BmnrEVEs could generate small interfering RNA (siRNA)-like RNAs, primary piRNA-like RNAs, or ping-pong-associated piRNAs. Functional analysis of BmToEVE and BmChuEVE3 further revealed distinct effects on larval development, cocoon traits, and reproduction. Notably, these two genes also differentially modulated infection by the noncognate viruses BmNPV and BmCPV. BmChuEVE3 knockdown enhanced the accumulation of both viruses, whereas BmToEVE knockdown markedly reduced BmNPV accumulation. These findings extend lepidopteran nrEVE research beyond genomic characterization and predicted small-RNA immunity, demonstrating that nrEVEs can acquire distinct physiological and antiviral functions.

## Materials and methods

### Silkworm strain and rearing conditions

The silkworm strain P50, a standard bivoltine strain producing yellowish green cocoons, was obtained from the Gene Resource Library of Domesticated Silkworm at Jiangsu University of Science and Technology, China. The P50 eggs used in this study were laid by female moths and immediately stored at 4°C for short-term preservation, with the storage period not exceeding 7 days. They were then subjected to a standard hydrochloric acid treatment to terminate diapause. Briefly, eggs were first acclimated at 15°C for 2 h and then immersed in hydrochloric acid with a specific gravity of 1.092 at 47.8°C for 5.5 min. Following standard sericultural incubation protocols [40], diapause-terminated eggs were maintained at 25°C under humid conditions until hatching. Larvae were reared under controlled environmental conditions, with temperatures maintained at 26–28°C, relative humidity at 70 ± 5%, and a photoperiod of 12 h light/12 h dark. Fresh mulberry leaves were supplied ad libitum throughout larval development. Larval growth and molting stages were monitored daily until the onset of cocoon spinning.

### Identification and genomic characterization of nrEVEs in *B.* *mori*

A comprehensive viral protein database was compiled from the NCBI non-redundant (nr) database (accessed September 2024) and supplemented with proteins from recently reported RNA viruses identified by LucaProt [41]. To identify nrEVEs in the *B. mori*, the reference genome (assembly GCF_030269925.1) was screened against this database using DIAMOND BLASTx v2.1.9 (E‑value ≤ 1 × 10^−5^). Putative nrEVE sequences were further validated by BLASTx against the NCBI nr database; hits with higher similarity to host insect proteins, retroviruses, or transposable elements were discarded. Validated nrEVEs were classified and named according to their best viral hits and named following a standardized nomenclature: Bm (host species) + viral family root name + numeric identifier. The numerical identifiers are used to distinguish between multiple nrEVEs derived from the same virus family.

Genomic locations were determined by mapping nrEVEs sequences back to the reference genome using BLASTn, requiring ≥ 80% query coverage and ≥80% nucleotide identity. Integration sites were visualized with the SilkMeta genome browser. Protein‑coding potential was assessed by ORF prediction using ORFfinder.

To assess cross-strain conservation, the predicted protein sequences of the identified BmnrEVEs were searched against 100 representative silkworm proteomes in the SilkMeta database. The dataset comprised 41 local strains, 15 improved strains, 30 genetic stocks, and 14 wild silkworm strains (Supplementary Table 1). For each BmnrEVE, the best BlastP hit in each strain was retained. A high-confidence homolog was defined by ≥90% amino-acid identity, ≥80% query coverage, and an *E*-value ≤ 1 × 10^−5^.

### Phylogenetic analysis of nrEVEs and related exogenous viruses

For phylogenetic inference, the amino acid sequences encoded by *B. mori* nrEVEs were aligned with related exogenous viruses using MAFFT v7. Maximum‑likelihood phylogenetic trees were reconstructed with IQ‑TREE v2.4.0 under the best‑fit substitution model selected by ModelFinder. Branch support was evaluated with 1,000 ultrafast bootstrap replicates.

### Nucleotide similarity between BmnrEVEs and prevalent silkworm viruses

To assess the potential homology between the identified BmnrEVEs and known viruses infecting *B. mori*, we performed a nucleotide similarity analysis using BLASTn. The genomic sequences of four prevalent *B. mori* viruses were downloaded from the NCBI Genome database: *B. mori infectious flacherie virus* (BmIFV; accessions GCF_001271195.1, GCF_000852185.1), *B. mori latent virus* (BmLV; GCF_002817955.1), *B. mori densovirus* (BmDNV; GCF_002819305.1, GCA_000861145.1, GCA_000908215.1), and BmCPV (GCA_023157015.1). Each nrEVE sequence was compared against these viral genomes using BLASTn with a dual threshold for a valid match set at ≥50% query coverage and ≥50% nucleotide identity.

### Characterization of BmnrEVEs-derived small RNAs and piRNA signatures

To determine whether the identified BmnrEVEs give rise to sRNAs, publicly available sRNA sequencing data from the P50 strain (BioProject PRJNA1001594) were analyzed. Raw reads were processed with Cutadapt v1.9 and FastQC v0.11.9 to remove adapters, low‑complexity reads, and low‑quality bases (Phred score < 20). High-quality reads ranging from 18 to 31 nucleotides in length were retained for downstream analyses. These filtered sRNAs were then aligned to the BmnrEVEs transcript sequences using Bowtie2 v2.5.4. Mapped reads were extracted with SAMtools v1.20, and their size distribution and strand specificity were analyzed with the viRome R package.

To further assess piRNA-associated features, BmnrEVE-mapped reads within the 26–30-nt size range were analyzed separately. Reads mapping to the positive and negative strands of each BmnrEVE were examined for nucleotide preferences characteristic of piRNA biogenesis. The frequency of uridine at the first nucleotide position (1 U) and adenine at the tenth position (10A) was calculated independently for each strand. Ping-pong amplification was evaluated from the relative positions of the 5' ends of oppositely oriented piRNA-sized reads. For reads mapped to the positive strand, the alignment start coordinate was defined as the 5' end, whereas the alignment end coordinate was used for reads mapped to the negative strand. Pairs of overlapping reads originating from opposite strands were identified, and the lengths of their 5'-to-5' overlaps were calculated. The distribution of overlap lengths was then examined for enrichment at 10 nt, a characteristic signature of ping-pong-dependent piRNA biogenesis [42]. The strength of the 10-nt overlap signal was evaluated using a Z-score relative to background overlap lengths excluding the 10-nt position. Sequence logos were additionally generated to visualize strand-specific nucleotide preferences. A pronounced 10-nt overlap together with complementary 1 U/10A biases was considered evidence of ping-pong-associated piRNA amplification [43,44]. Loci showing strand-biased, 1 U-enriched piRNA-sized sRNAs without a clear 10-nt overlap were classified as exhibiting predominantly primary piRNA-like processing [43,44].

### Assessment of BmnrEVEs transcription

To assess whether the identified BmnrEVEs are transcriptionally active in the silkworm P50 strain, publicly available RNA-seq datasets (BioProject: PRJNA559726) were analyzed. When available, datasets encompassing multiple developmental stages, including larval, pupal, and adult stages, were selected to provide a broad survey of nrEVE transcription. Raw sequencing reads were first subjected to quality assessment using FastQC v0.11.9, followed by adapter and low-quality base trimming with Trimmomatic v0.40. The processed reads were then assembled de novo using Trinity v2.9.0. To identify nrEVE-derived transcripts, the assembled contigs were aligned against the BmnrEVEs using BLASTn, with an E-value cutoff of 1e-5.

To experimentally validate BmnrEVEs transcription, reverse transcription PCR (RT-PCR) was performed. Total RNA was extracted from pooled larval samples encompassing all five instars using TRIzol™ Reagent (Thermo Fisher Scientific, 15596026CN). First-strand cDNA synthesis was carried out using PrimeScript RT Master Mix (Takara, RR036Q). PCR amplification was conducted with BmnrEVEs-specific primers designed to span the full length of selected nrEVE transcripts (primer sequences are listed in Supplementary Table 2). Amplified products were confirmed by Sanger sequencing, and the validated BmnrEVE sequences are provided in Supplementary Table 3.

### Spatiotemporal expression analysis of BmnrEVEs

To characterize the spatiotemporal transcriptional profiles of BmnrEVEs, their relative transcript abundance was examined across larval development and different tissues of the P50 strain using quantitative real-time PCR (qPCR). For developmental profiling, whole larvae were collected throughout the five larval instars (L1–L5) at representative developmental transitions, including molting, post-molt feeding, and the first day of the wandering stage. For tissue-specific profiling, the head (HD), epidermis (EP), hemolymph (HL), fat body (FB), midgut (MG), silk gland (SG), testis (TS), and ovary (OV) were collected from larvae on the first day of the wandering stage. Three independent biological replicates were prepared for each developmental stage or tissue, with each replicate consisting of pooled samples from five larvae or the corresponding tissues.

Total RNA was isolated using TRIzol Reagent (ThermoFisher, 15596026CN) and treated with DNase I (Takara, 2270A) to remove residual genomic DNA. First-strand cDNA was synthesized from 1 µg of purified RNA with PrimeScript RT Master Mix (Takara, RR036Q). qPCR was performed using TB Green Premix Ex Taq II (TaKaRa, CN830S) on a LightCycler 96 Real-Time PCR System. *BmActin3* was used as the internal reference gene, and relative transcript abundance was calculated using the 2^(−ΔΔCt) method. For each BmnrEVE, the sample showing the lowest transcript abundance within the corresponding developmental or tissue series was used as the calibrator and assigned a relative expression value of 1. All primer sequences are listed in Supplementary Table 2.

### Knockdown of BmnrEVEs mediated by a dsRNA/nanoparticle complex

Based on their distinct temporal expression patterns and sRNA profiles, BmToEVE and BmChuEVE3 were selected for functional investigation via RNAi. DNA fragments corresponding to BmToEVE and BmChuEVE3 were amplified by PCR using primers containing T7 promoter sequences (Supplementary Table 2). In parallel, the green fluorescent protein (GFP) gene was amplified as a control. Double-stranded RNA (dsRNA) was synthesized from the PCR products using the T7 High Yield RNA Transcription Kit (Promega, P1700).

To facilitate oral dsRNA delivery in *B. mori*, a star polycation (SPc; provided by China Agricultural University) was used as a nanocarrier according to a previously established method [45]. Gene-specific dsRNA was mixed with SPc at a 1:1 mass ratio (w/w) in double-distilled water (ddH_2_O; Sangon Biotech, B501005-0500) [45]. The final concentrations of both dsRNA and SPc were adjusted to 500 ng/μL. The mixture was gently mixed and incubated at 25°C for 15 min to allow formation of dsRNA/SPc complexes. Under aqueous conditions, dsRNA electrostatically self-assembled with SPc to generate stable dsRNA/SPc nanoparticles. The leaf-surface feeding conditions were adapted and empirically optimized for *B. mori*. Specifically, 4 μL of the dsRNA/SPc formulation was evenly applied to a 1 × 1 cm section of fresh mulberry leaf. This application volume was selected because it could be retained and fully absorbed by the leaf surface without appreciable runoff, while the small leaf section facilitated rapid and complete consumption by the larvae. The leaves were allowed to fully absorb the solution for 4 hours before being fed to silkworm larvae. At 24 and 48 hours post-feeding (hpf), five larvae were randomly selected from each treatment group for the detection of target gene expression levels. Five biological replicates were set up for each treatment to ensure the reliability and reproducibility of the experimental results. For the control group, 4 μL of dsGFP/SPc solution was uniformly sprayed onto the surface of mulberry leaves.

### Phenotypic assessment following BmToEVE and BmChuEVE3 knockdown

To characterize the biological functions of BmToEVE and BmChuEVE3, a comparative phenotypic analysis was performed following SPc-mediated RNAi. Based on their developmental expression profiles, RNAi treatment was initiated 48 h before the respective high-expression stages of the two BmnrEVEs. This design ensures effective suppression of target gene expression at their biologically active stages, thus maximizing the likelihood of capturing distinct phenotypic alterations associated with gene depletion. For BmToEVE knockdown, larvae were first provided with mulberry leaves treated with 4 μL of dsBmToEVE/SPc formulation (500 ng/μL dsRNA) on day 2 of the second instar (L2D2), prior to the post-molt feeding phase of the third instar. BmChuEVE3 knockdown was initiated later, on day 3 of the fourth instar (L4D3), before feeding resumed in the fifth instar. The dsRNA/SPc treatments were repeated at 48-h intervals throughout the subsequent feeding period. Control larvae received an equivalent dose of dsGFP/SPc prepared and administered under the same conditions.

Larval growth and development were monitored throughout the experiment. Males and females of the P50 strain cannot be reliably distinguished by external morphology during the larval stages without invasive examination. Therefore, larval sex was not determined, and developmental traits were assessed in mixed-sex populations. The duration of each larval instar was recorded, and the mean developmental duration was calculated as Σ(f_i_ × x_i_)/N, where f_i_ represents the number of larvae with a developmental duration of x_i_ days and N represents the total number of larvae examined. Larval body weight was measured at 24- or 48-h intervals. Thirty larvae constituted each biological replicate, and body weight was recorded in groups of five larvae; the resulting measurements were averaged for each replicate. Cocooning was monitored daily, and mean cocooning time was calculated as ∑(n_i_ × t_i_) / N, where n_i_ represents the number of larvae initiating cocoon formation on day t_i_. After cocoon formation, sex was determined and cocoon traits were evaluated separately for males and females to account for sex-dependent variation in these traits. Whole-cocoon weight, cocoon shell weight, maximum cocoon length, and maximum cocoon width were measured. The cocoon shell ratio was calculated as (shell weight / total weight) × 100%. Male and female cocoons were analyzed separately to account for sex-dependent differences in cocoon traits. For fecundity assessment, newly emerged male and female moths were paired at a 1:1 ratio. Fecundity was determined as the total number of eggs laid by each mating pair. Eggs were incubated at 25°C and 75% relative humidity. Hatchability was evaluated by counting hatched larvae and unhatched embryos.

All experiments included three biological replicates. For body weight, developmental duration, mean cocooning time, and cocoon shell ratio, each replicate used 30 individuals. For fecundity analysis, 12 mating pairs were examined per replicate.

### Yeast two-hybrid screening for interacting proteins of BmToEVE and BmChuEVE3

To identify proteins interacting with BmToEVE and BmChuEVE3, full‑length coding sequences of both genes were cloned into the pGBKT7 bait vector. A *B. mori* P50 cDNA prey library was constructed in pGADT7 using the Nuclear/Membrane Yeast Two‑Hybrid Library Construction Kit (GeneCREATE, JKR23009HM). Bait and prey plasmids were co‑transformed into yeast strain AH109 (Clontech) via the LiAc/PEG method (Coolaber, YT0002/YT0001). Transformants were plated on quadruple dropout medium (SD/–Trp/–Leu/–His/–Ade; Takara, 630,464) containing X-α-gal (Takara, 630,463) to select for positive colonies.

Candidate interacting proteins were initially identified by performing colony PCR on individual positive colonies using the Yeast Colony Rapid Detection Kit (Nanjing Ruiyuan Biotechnology, RY8001). Prey inserts were amplified and sequenced by the Sanger method. Sequences were compared to public databases via BLAST, and redundant clones were removed to generate a non-redundant list of candidate interactors.

In parallel, all positive colonies were pooled to enable comprehensive identification of interacting proteins and assessment of their relative abundance. The resulting reads were mapped to the *B. mori* reference genome using HISAT2 v2.2.1. Candidate genes were further annotated using BLAST and functional databases, including AnimalTFDB for transcription factor identification and hmmscan for kinase domain prediction. Only matches with ≥85% sequence identity and supported by at least 20 sequencing reads were retained for downstream analysis.

### Transcriptome analysis following BmToEVE and BmChuEVE3 knockdown

To assess the transcriptional changes associated with BmnrEVE knockdown in the absence of viral infection, RNA sequencing was performed on control and knockdown larvae. Samples were collected at the developmental stages when knockdown-associated phenotypes were assessed, enabling direct comparison between transcriptional and phenotypic changes. Larvae were treated with dsRNA/SPc complexes targeting BmToEVE or BmChuEVE3, together with the corresponding control treatments, as described above. Samples were collected during the resumption of feeding in the third instar for BmToEVE and in the fifth instar for BmChuEVE3. Four independent biological replicates were analyzed for each condition, with five larvae pooled per replicate.

Following sample collection, total RNA was extracted using TRIzol (ThermoFisher, 15596026CN) reagent following the manufacturer’s instructions. RNA samples that passed quality assessment were submitted to LC-Bio Technology Co., Ltd. (Hangzhou, China) for library preparation. Libraries were sequenced on an Illumina NovaSeq X Plus platform using a paired-end 150 bp strategy. Raw reads were processed with Cutadapt v1.9 to remove adapter sequences, poly-A/-G tails, reads containing > 5% unknown nucleotides (N), and low-quality reads (>20% of bases with Q-value ≤ 20). The quality of the resulting clean reads was assessed using FastQC v0.11.9, including evaluation of Q20 and Q30 scores and GC content. Clean reads were subsequently aligned to the P50 reference genome using HISAT2 v2.2.1. Gene expression was quantified with StringTie v2.1.6 and Ballgown, normalized as fragments per kilobase of transcript per million mapped reads (FPKM). Differential expression analysis was performed using DESeq2 and edgeR. Genes with a false discovery rate (FDR) <0.05 and an absolute fold change ≥ 2 were classified as differentially expressed genes (DEGs). Functional enrichment analysis of DEGs was conducted using Gene Ontology (GO) and Kyoto Encyclopedia of Genes and Genomes (KEGG) pathway analyses based on a hypergeometric test. GO terms or KEGG pathways with a *p*-value < 0.05 were considered significantly enriched.

### Viral infection assays and functional validation

To investigate virus-induced changes in BmToEVE and BmChuEVE3 expression, larvae were orally inoculated with BmNPV or BmCPV on day 1 of the fifth instar (L5D1). This early fifth-instar stage, commonly used for experimental viral infection in *B. mori*, was selected to standardize oral exposure and minimize developmental variation among treatments. For BmNPV infection, mulberry leaves (4 × 4 cm) were treated with 3 µL of viral suspension containing 1 × 10^9^ occlusion bodies (OBs)/mL. The suspension was evenly spread on the leaf surface and air-dried before feeding. For BmCPV infection, 1 µL of viral suspension containing 1 × 10^6^ polyhedra/µL was similarly applied to a mulberry leaf. Larvae were housed individually to ensure complete consumption of the virus-treated leaves. Mock-infected controls were provided with mulberry leaves treated with the corresponding volume of PBS (Servicebio, G4202). The time at which the virus- or PBS-treated leaves were provided was designated as 0 hpi. After inoculation, larvae were maintained on fresh mulberry leaves under standard rearing conditions. To analyze BmnrEVEs expression following viral infection, larvae were collected at 3 and 6 days post-infection (dpi). Total RNA was extracted using TRIzol reagent (ThermoFisher, 15596026CN), and the relative mRNA levels of BmToEVE and BmChuEVE3 were quantified by qPCR using the housekeeping genes and method described above. Four biological replicates were performed, each consisting of 15 larvae.

To investigate the potential antiviral role of these BmnrEVEs, larvae at the L4D3 stage were first fed mulberry leaves treated with dsRNA/SPc complexes (4 µL, 500 ng/µL) targeting BmToEVE, BmChuEVE3, or GFP (control). Larvae were subsequently orally inoculated with BmNPV or BmCPV at L5D1, as described above. To sustain the knockdown efficiency, a second administration of the respective dsRNA/SPc complex was performed on L5D2. Whole larvae were collected at 3 and 6 dpi for viral-load quantification. For each BmNPV-infected biological replicate, total DNA and total RNA were extracted in parallel from the same biological sample. Total DNA was used for viral-load quantification, whereas the matched RNA was used for the transcriptomic analyses described below. Relative BmNPV DNA load was quantified by qPCR using primers targeting the viral *gp41* gene, with the *B. mori GAPDH* genomic locus used as an internal reference. Relative viral DNA abundance was calculated using the 2^(−ΔΔCt) method, with the dsGFP-treated BmNPV group at the corresponding time point used as the calibrator. For BmCPV-infected samples, total RNA was extracted from the corresponding whole larvae and reverse-transcribed into cDNA. Relative BmCPV RNA abundance was determined by RT-qPCR using primers targeting the viral S2 segment (GenBank: AY496445.1; Supplementary Table 2) and normalized to the reference gene described above. RNA from the same biological samples was used for transcriptome sequencing.

### Identification of host genes involved in BmnrEVE-mediated viral regulation

To characterize host transcriptional responses to BmChuEVE3 and BmToEVE during viral infection, we performed RNA-seq on samples from the knockdown and infection experiments described above. Larvae were treated with dsRNA/SPc at L4D3, orally inoculated with BmNPV or BmCPV at L5D1, and received a second dsRNA/SPc treatment at L5D2. Based on the viral-load phenotypes described above, matched samples collected at 3 dpi were used for transcriptomic analysis of BmChuEVE3, whereas samples collected at 6 dpi were used for BmToEVE. For BmChuEVE3, the 3-dpi transcriptomic analysis included the time-matched dsGFP/SPc + PBS, dsGFP/SPc + virus, and dsBmChuEVE3/SPc + virus groups for each viral infection model. For BmToEVE, the corresponding 6-dpi analysis included dsGFP/SPc + PBS, dsGFP/SPc + virus, and dsBmToEVE/SPc + virus groups. Thus, all transcriptomic comparisons were performed within the same viral infection model and at the same sampling time point.

High‑quality RNA samples were submitted to Hangzhou LC‑Bio Technology Co., Ltd. for library preparation. Sequencing was performed on an Illumina NovaSeq™ X Plus platform (150‑bp paired‑end). The bioinformatics workflow for read quality control, genome alignment, gene expression quantification, differential expression analysis, and functional enrichment analysis was identical to that described in the previous section. Four biological replicates were included for each treatment group, with each replicate consisting of five pooled larvae.

To identify host genes potentially involved in BmToEVE- and BmChuEVE3-mediated regulation of viral replication, a stepwise comparative transcriptomic analysis was conducted independently for the BmNPV and BmCPV infection datasets. First, virus‑induced DEGs (DEGs‑virus) were identified by comparing each virus‑infected group to its mock‑infected control (dsGFP/SPc + BmNPV vs dsGFP/SPc + PBS for BmNPV; dsGFP/SPc + BmCPV vs dsGFP/SPc + PBS for BmCPV). These DEGs represent the core host transcriptional responses to each viral infection. Next, DEGs associated with nrEVE knockdown under infection (DEGs‑dsBmnrEVEs) were identified by comparing each nrEVE‑knockdown group to the dsGFP control within the same viral context (dsBmToEVE/SPc + Virus vs dsGFP/SPc + Virus, and dsBmChuEVE3/SPc + Virus vs dsGFP/SPc + Virus, separately for BmNPV and BmCPV). These comparisons pinpoint host genes whose infection-responsive expression is functionally linked to the presence of either BmToEVE or BmChuEVE3. Candidate host genes were defined as those present in the DEGs-virus set but absent from the corresponding DEGs-dsBmnrEVEs set, or those exhibiting substantially altered fold changes between the two comparisons. These genes were inferred to be potentially involved in the specific or cooperative regulatory roles of BmToEVE and BmChuEVE3 in modulating host responses and viral replication under distinct viral infection contexts.

### Statistical analysis

Statistical analyses were performed using GraphPad Prism v 9.5. For comparisons between two groups, two-tailed Student’s *t*-tests were utilized. Comparisons across multiple groups were conducted using one-way analysis of variance (ANOVA) followed by Tukey’s honestly significant difference (HSD) post hoc test. A probability (*p*) value of less than 0.05 was considered statistically significant. All data are expressed as the mean ± standard error of the mean (SEM) of independent biological replicates.

## Results

### Diverse nrEVEs identified in the silkworm genomes

**Figure 1.**
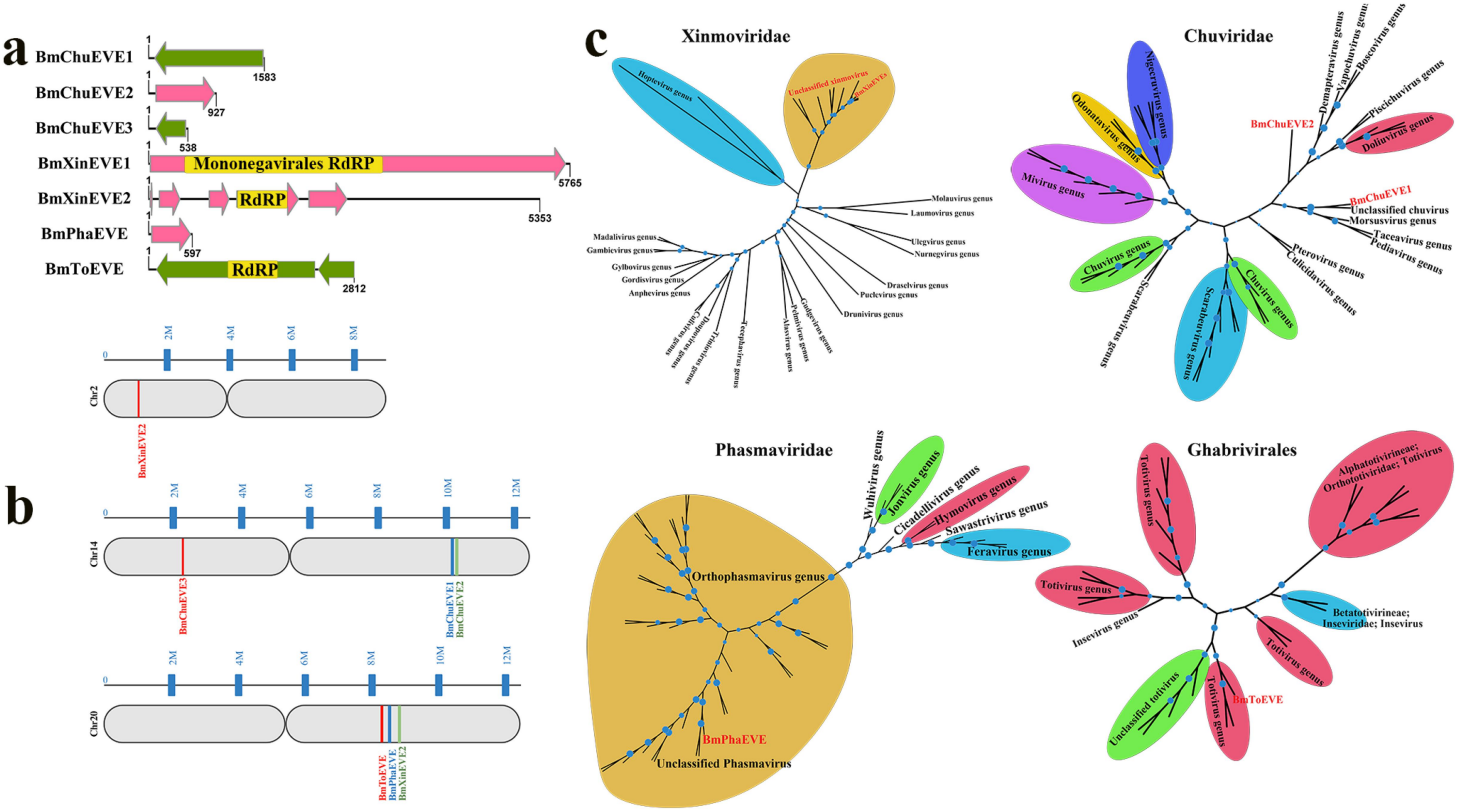
Genomic characterization and phylogenetic analysis of nrEVEs in *B. mori*. (a) Predicted ORFs of the seven nrEVEs identified in the *B. mori* P50 genome. Arrowheads point in the direction of transcription. Numbers indicate the nucleotide length of each nrEVE. Annotated conserved domains are highlighted in yellow. (b) Chromosomal landscape of nrEVEs in the silkworm genome. (c) Phylogenetic analysis of silkworm nrEVEs and related exogenous viruses based on predicted amino acid sequence. Maximum likelihood trees were inferred based on GP (*Phasmaviridae*) or RdRP (other families). In the tree, BmnrEVEs are highlighted in red, and branch support is indicated by solid blue circles, with larger circles corresponding to higher bootstrap values. BmChuEVE3 was excluded because its predicted amino acid sequence was too short for reliable alignment.

Genome-wide screening of the *B. mori* P50 assembly identified seven nrEVEs originating from four RNA virus families, including three *Chuviridae*-derived elements (BmChuEVE1–3), two *Xinmoviridae*-derived elements (BmXinEVE1–2), one *Phasmaviridae*-derived element (BmPhaEVE), and one *Totiviridae*-derived element (BmToEVE) (Table 1). Six of these elements were derived from negative-sense RNA viruses, whereas BmToEVE originated from a double-stranded RNA virus. Coding-potential analysis revealed distinct ORF organizations among the seven nrEVEs (Figure 1(a)). Five showed homology to viral non-structural genes encoding RNA-dependent RNA polymerases (RdRPs), whereas BmPhaEVE and BmChuEVE3 showed homology to viral structural protein genes encoding glycoproteins (GPs) (Table 1). BmChuEVE1, BmChuEVE2, BmChuEVE3, BmXinEVE1, and BmPhaEVE each contained a single major ORF, whereas BmXinEVE2 contained multiple shorter ORFs and BmToEVE contained two reverse-oriented ORFs (Figure 1(a)). Conserved-domain analysis further revealed that BmXinEVE1, BmXinEVE2, and BmToEVE each retained a recognizable RdRP domain (Figure 1(a)). In contrast, no recognizable conserved protein domains were detected in BmChuEVE1, BmChuEVE2, BmChuEVE3, or BmPhaEVE, despite their sequence homology to the corresponding viral protein-coding regions (Figure 1(a)).

**Table 1.** Summary of nrEVEs identified in the *Bombyx mori* P50 genome.

nrEVEs name	Viral family	Nucleic acid type		Chromosomal location	Encoded protein	Best BLASTx hit	Amino acid identity (%)
BmChuEVE1	*Chuviridae*		(-)ssRNA	Chr 14	RdRP	Chuviridae sp.	65.45%
BmChuEVE2					RdRP	Chuviridae sp.	48.28%
BmChuEVE3					GP	Orthornavirae sp.	68.52%
BmXinEVE1	*Xinmoviridae*			Chr 20	RdRP	Nymphalis C-aureum xinmovirus 1	79.86%
BmXinEVE2				Chr 2	RdRP	Nymphalis C-aureum xinmovirus 1	74.26%
BmPhaEVE	*Phasmaviridae*			Chr 20	GP	Mothra virus	38.65%
BmToEVE	*Totiviridae*		dsRNA	Chr 20	RdRP	Totivirus sp.	45.18%

To assess whether the nrEVEs identified in P50 are conserved across silkworm genetic backgrounds, we further searched their predicted proteins against 100 annotated genomes available in SilkMeta (Supplementary Table 1). The seven BmnrEVEs exhibited markedly different cross-strain distributions. High-confidence homologs of BmXinEVE1, BmToEVE, BmChuEVE1, BmChuEVE2, and BmPhaEVE were detected in 65, 62, 60, 49, and 34 of the 100 genomes, respectively (Supplementary Table 4). By comparison, a BmXinEVE2 homolog was detected in only one strain, whereas no BmChuEVE3 homolog was identified in any strain other than P50. These results reveal marked variation among strains in the conservation of BmnrEVEs.

Sequence similarity searches showed that the predicted amino acid identities between nrEVEs and their closest viral homologs ranged from 38.65% to 79.86%, with nrEVEs from *Xinmoviridae* consistently exhibiting higher similarity values (>70%, Table 1). Interestingly, some nrEVEs shared identical best BLASTx hits. For example, two *Chuviridae* nrEVEs (BmChuEVE1 and BmChuEVE2) matched the same unclassified chuvirus based on RdRp similarity. However, the corresponding sequences mapped to non-overlapping viral ORFs, suggesting that they may have originated from the same or closely related ancestral virus. Likewise, BmXinEVE1 and BmXinEVE2, shared the same top BLAST hit (*Nymphalis C-aureum xinmovirus 1*). Notably, their predicted protein sequences shared 73.39% amino acid identity, supporting a close evolutionary relationship between the two nrEVEs and suggesting that they may have originated from a common ancestral viral sequence.

Notably, BmnrEVEs were not randomly distributed in the *B. mori* genome and exhibited a distinct clustering pattern on specific chromosomes. All three *Chuviridae*-derived elements (BmChuEVE1–3) were inserted on chromosome 14, although separated by substantial genomic distances, indicating multiple independent insertion events (Figure 1(b)). Three additional nrEVEs (BmXinEVE1, BmPhaEVE, BmToEVE) were all integrated into chromosome 20, whereas BmXinEVE2 was located on chromosome 2 (Figure 1(b)). Such non-uniform chromosomal patterns may reflect preferential hotspots for viral integration.

A critical finding was the lack of significant nucleotide or amino acid sequence similarity between any of the identified nrEVEs and the four known, currently circulating RNA viruses (BmCPV, BmDNV, BmIFV, BmLV) that infect silkworms or its cell lines. This lack of detectable homology suggests integration events either from ancient viral lineages that are no longer circulating, or from recent, yet-unsampled viruses that remain undiscovered in natural *B. mori* populations.

To clarify the evolutionary relationships between BmnrEVEs and extant viruses, we constructed ML phylogenetic trees based on GP and RdRP amino acid sequences. The resulting trees clear placed most nrEVEs within or near specific viral lineages (Figure 1(c)). BmPhaEVE robustly clustered within the established genus *Orthophasmavirus*, alongside two other currently unclassified phasmaviruses (Figure 1(c)). Similarly, BmToEVE formed a well-supported clade with members of the genus *Totivirus*, solidifying its classification within this group (Figure 1(c)). The two BmXinEVEs formed a highly supported monophyletic clade with other recently identified, yet unclassified, xinmoviruses derived from insects (Figure 1(c)). This distinct clustering suggests that these nrEVEs, along with their viral relatives, may represent a novel, cohesive lineage within the *Xinmoviridae* family, potentially warranting future taxonomic consideration as a new genus. In contrast to the clear placements above, the phylogenetic position of the *Chuviridae*-derived elements remained less definitive. BmChuEVE1 and BmChuEVE2 did not form a stable cluster with any currently classified viral genera within *Chuviridae* (Figure 1(c)). This indicates that their ancestral viruses likely represent a distinct and potentially uncharacterized evolutionary lineage within this diverse family. It is noteworthy that BmChuEVE3 was excluded from the phylogenetic analysis due to its insufficiently short predicted GP protein sequence, which prevented a reliable alignment.

### BmnrEVEs are transcriptionally active and exhibit distinct spatiotemporal expression patterns

To assess the transcriptional activity of the identified BmnrEVEs, we first surveyed publicly available RNA-seq datasets from the P50 strain. Transcripts corresponding to all seven BmnrEVEs were detected, indicating that these genomic elements are actively transcribed. We next independently validated their transcription by RT-PCR using cDNA prepared from P50 RNA and multiple primer pairs spanning each nrEVE. Nearly full-length transcripts were successfully recovered for all seven nrEVEs (Supplementary Table 3), further confirming their transcription in vivo.

**Figure 2.**
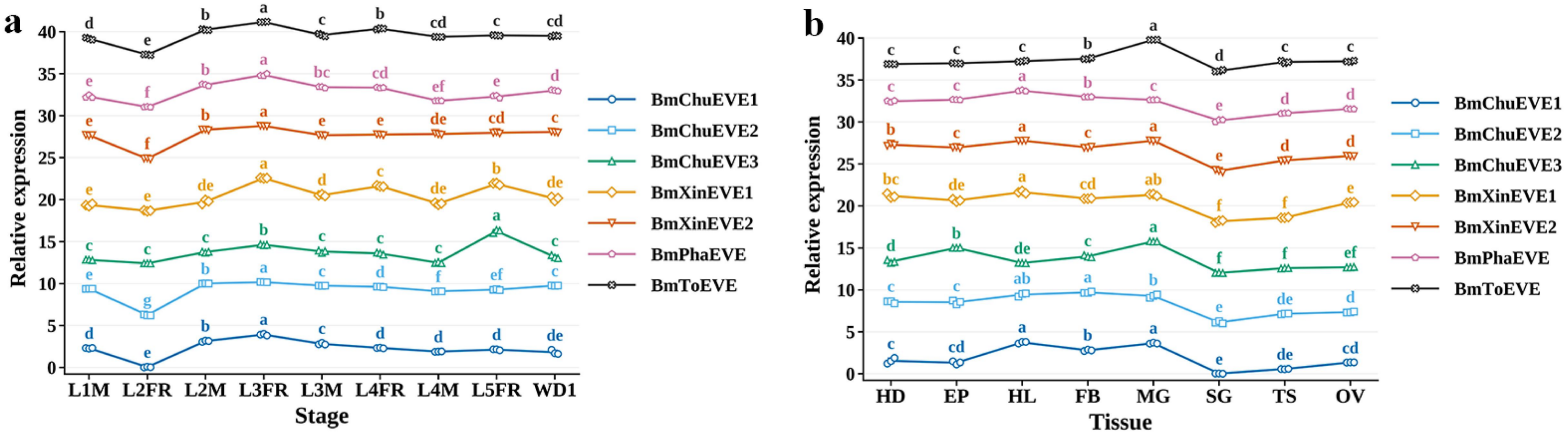
Transcriptional dynamics of the seven nrEVEs across developmental stages and tissues in *B. mori*. (a) Developmental expression profiles of nrEVEs across selected larval stages and the first day of the wandering stage. M, molting stage; FR, feeding resumption (~24 h after ecdysis); WD1, day 1 of the wandering stage. (b) Tissue-specific expression patterns of nrEVEs at WD1. hd, head; EP, epidermis; HL, hemolymph; FB, fat body; MG, midgut; SG, silk gland; ts, Testis; OV, ovary. Relative expression levels were calculated using the 2^^^(-ΔΔCt) method with BmActin3 as the reference gene. For visualization, values were log2(x + 1)-transformed, min–max scaled within each nrEVE, and vertically offset to separate overlapping trajectories; these transformed values were not used for statistical inference. Points represent three biological replicates, each comprising a pool of five larvae. For each nrEVE, statistical comparisons among developmental stages (a) or tissues (b) were performed on the original, untransformed relative-expression values using one-way ANOVA followed by Tukey’s HSD multiple-comparison test. Different lowercase letters denote significant differences (Tukey-adjusted *p* < 0.05); groups sharing at least one letter are not significantly different. Source data, including the original relative-expression values, visualization-transformed values, and statistical analyses, are available in figshare.

We next characterized the developmental expression profiles of the seven BmnrEVEs. Developmental profiling revealed distinct expression dynamics among the elements (Figure 2(a)). BmChuEVE1, BmChuEVE2, BmToEVE, BmPhaEVE, and BmXinEVE2 showed increased transcript abundance during the post-ecdysial feeding-resumption phase of the third instar, approximately 24 h after ecdysis (Figure 2(a)). However, comparable increases were not consistently observed at the corresponding stages of subsequent instars. BmChuEVE3 reached its expression peak during the analogous feeding resumption phase in the 5th instar (Figure 2(a)). In contrast, BmXinEVE1 exhibited a recurrent temporal pattern, with elevated transcript abundance during the post-ecdysial feeding phases of the third, fourth, and fifth instars (Figure 2(a)). Thus, BmnrEVEs displayed markedly distinct developmental profiles, ranging from instar-restricted expression maxima to recurrent post-ecdysial elevation.

Tissue profiling at the wandering stage further revealed broad but nonuniform expression of BmnrEVEs (Figure 2(b)). Transcripts were detected across multiple tissues, with comparatively higher abundance in the MG, EP, and HD. The relatively high expression of BmnrEVEs in these tissues suggests potential tissue-specific functions, including roles in host–virus interactions. Together, these analyses show that BmnrEVEs are not uniformly expressed throughout silkworm development. Instead, their expression varies among developmental stages and tissues in a locus-specific manner.

### BmnrEVEs generate both siRNAs and ping-pong-amplified piRNA-like small RNAs

**Figure 3.**
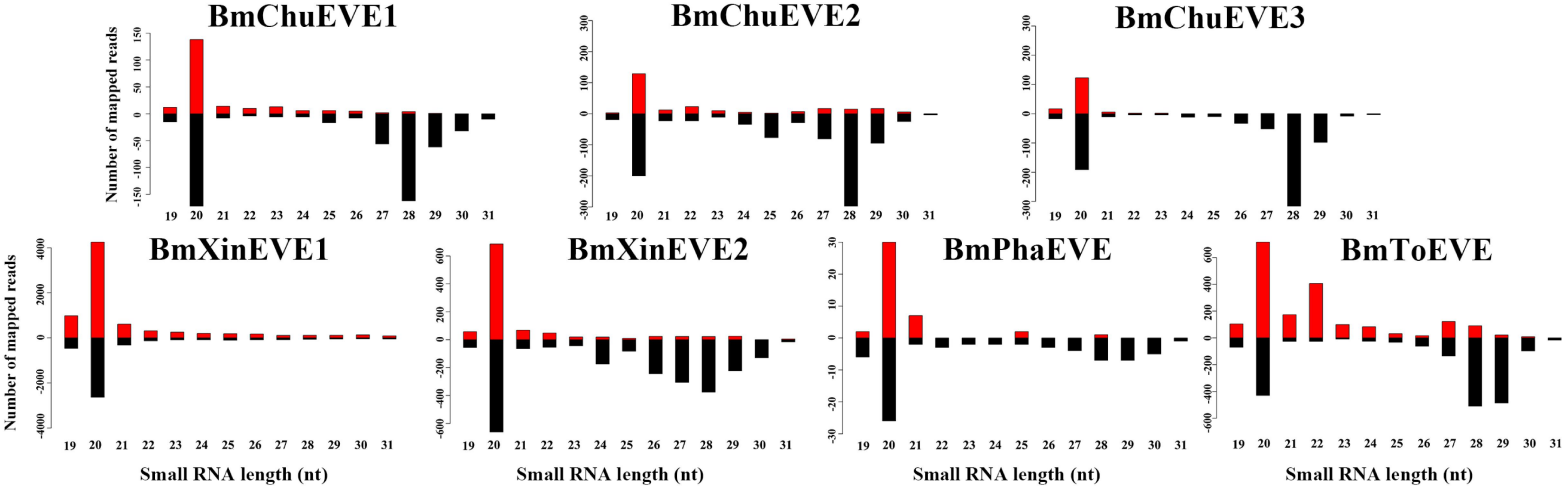
Length and strand distributions of nrEVE-derived small RNAs in *B. mori*.

**Figure 4.**
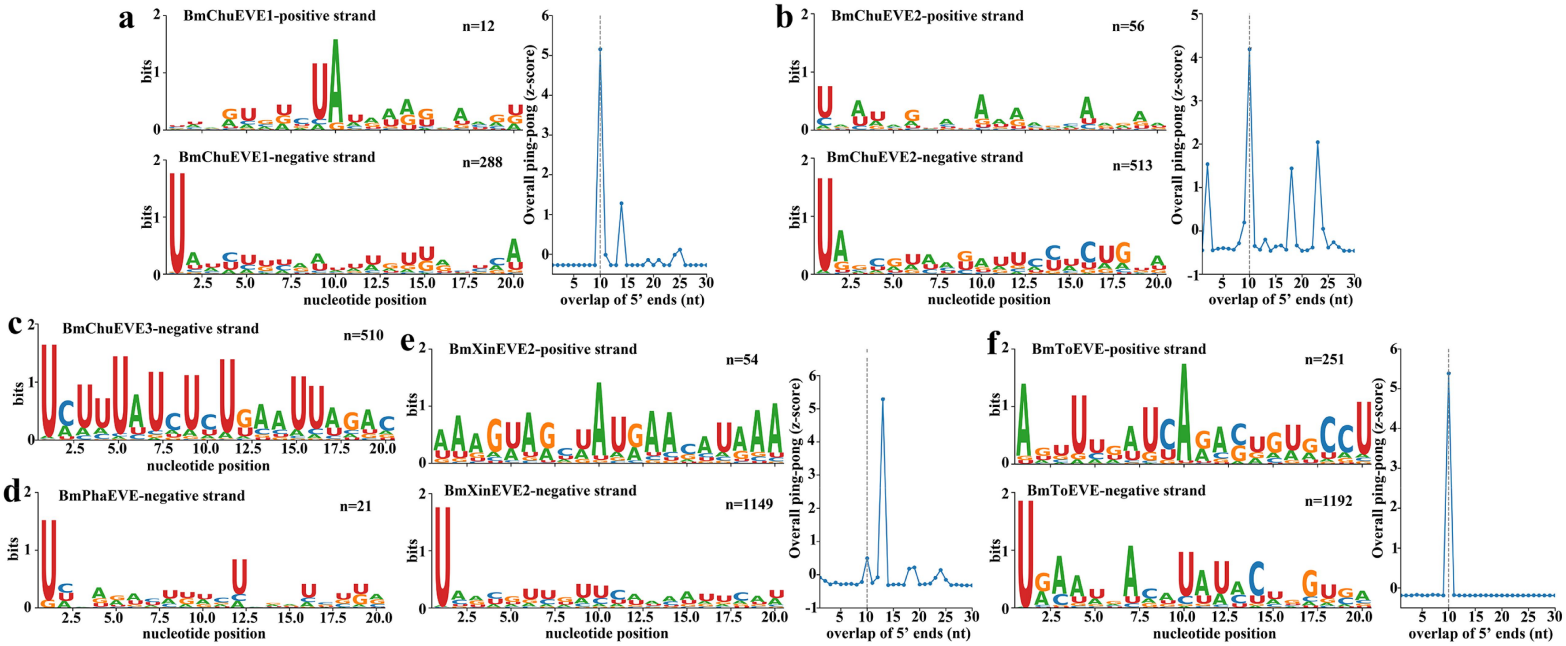
Strand-specific nucleotide biases and ping-pong signatures of nrEVE-derived piRnas. sequence logos show nucleotide preferences within the first 20 positions of positive- and/or negative-strand reads. The letter height represents nucleotide information content in bits, and n indicates the number of reads included in each sequence-logo analysis. Where sufficient reads were available from both orientations, ping-pong signatures were evaluated from the Z-score distribution of 5'-end overlaps between oppositely oriented small RNAs. The vertical dashed line denotes the canonical 10-nt overlap. (a, b) BmChuEVE1 and BmChuEVE2 showed a strong 1 U bias in negative-strand reads and a prominent 10-nt 5'-end overlap. (c, d) BmChuEVE3 and BmPhaEVE showed predominantly negative-strand, 1 U-biased populations; overlap analysis was not informative because of insufficient oppositely oriented reads. (e) BmXinEVE2 showed a strong negative-strand 1 U bias, whereas the major 5'-end overlap peak occurred outside the canonical 10-nt position. (f) BmToEVE showed a negative-strand 1 U bias, a positive-strand 10A-associated bias, and a pronounced 10-nt 5'-end overlap.

In various animals, including insects, nrEVEs have been widely reported to give rise to piRNAs, potentially serving as a memory bank for adaptive immunity against cognate viruses [35,46]. To determine whether the BmnrEVEs enter endogenous small-RNA pathways, we characterized the size distribution, strand polarity, nucleotide composition, and 5'-end overlap of sRNAs mapping to each nrEVE (Figure 3 and 4). BmnrEVEs exhibited markedly different sRNA profiles. All three BmChuEVEs generated a prominent 20-nt sRNA peak together with a broader class of longer reads, with the greatest abundance at approximately 28 nt (Figure 3). Whereas the 20-nt reads were recovered from both genomic strands, the piRNA-sized reads showed a pronounced strand bias, with most mapping to the negative strand. BmPhaEVE displayed a similar, although substantially less abundant, profile. The bidirectional 20-nt reads are consistent with siRNA-like processing, particularly given that *Dicer*-2-dependent antiviral sRNAs in *B. mori* predominantly accumulate at approximately 20 nt [47]. By contrast, the longer, strongly strand-biased reads exhibit features characteristic of piRNA-like sRNAs [43].

Positive-strand reads are shown above the horizontal axis in red, whereas negative-strand reads are shown below the axis in black. Strand orientation is defined relative to the corresponding nrEVE reference sequence. The y-axis represents the number of mapped reads. This analysis utilized publicly available small RNA-seq data from the *B. mori* P50 strain whole-larva samples (PRJNA1001594).

We next examined whether the piRNA-sized sRNAs displayed nucleotide and 5'-end overlap signatures diagnostic of piRNA biogenesis. Negative-strand sRNAs derived from BmChuEVE1 showed a pronounced 1 U bias, whereas the corresponding positive-strand population was enriched for adenine at nucleotide 10 (10A) (Figure 4(a)). Consistent with these complementary nucleotide preferences, oppositely oriented BmChuEVE1 sRNAs exhibited a sharp 10-nt overlap between their 5' ends (Figure 4(a)), a hallmark of ping-pong amplification. A similarly prominent 10-nt overlap was observed for BmChuEVE2-derived sRNAs (Figure 4(b)), indicating that at least two of the three chuvirus-derived nrEVEs are processed through a ping-pong-associated piRNA pathway. In contrast, BmChuEVE3 and BmPhaEVE produced strongly strand-biased piRNA-sized sRNAs with a pronounced 1 U preference but without detectable evidence of a canonical 10-nt ping-pong overlap (Figure 4(c,d)). These profiles are therefore more consistent with predominantly primary piRNA-like biogenesis than with extensive secondary amplification. BmXinEVE2 also generated piRNA-sized reads with a strong 1 U bias on the negative strand (Figure 4(e)). However, these reads lacked the characteristic 10-nt 5'-end overlap, providing no clear evidence for canonical ping-pong amplification at this locus (Figure 4(e)). In contrast, BmToEVE showed a pronounced ping-pong signature (Figure 4(f)). Negative-strand sRNAs exhibited a strong 1 U bias, whereas positive-strand sRNAs were enriched for adenine at position 10 (10A). Moreover, oppositely oriented sRNAs displayed a prominent 10-nt 5'-end overlap (Figure 4(f)), a hallmark of ping-pong amplification. Together, these findings reveal marked locus-specific differences in nrEVE-derived sRNA biogenesis in *B. mori*. Individual nrEVEs appear to undergo distinct modes of processing, ranging from siRNA-like processing and strand-biased primary piRNA production to ping-pong-associated piRNA amplification.

### BmToEVE and BmChuEVE3 exert divergent essential roles in silkworm development and reproduction

**Figure 5.**
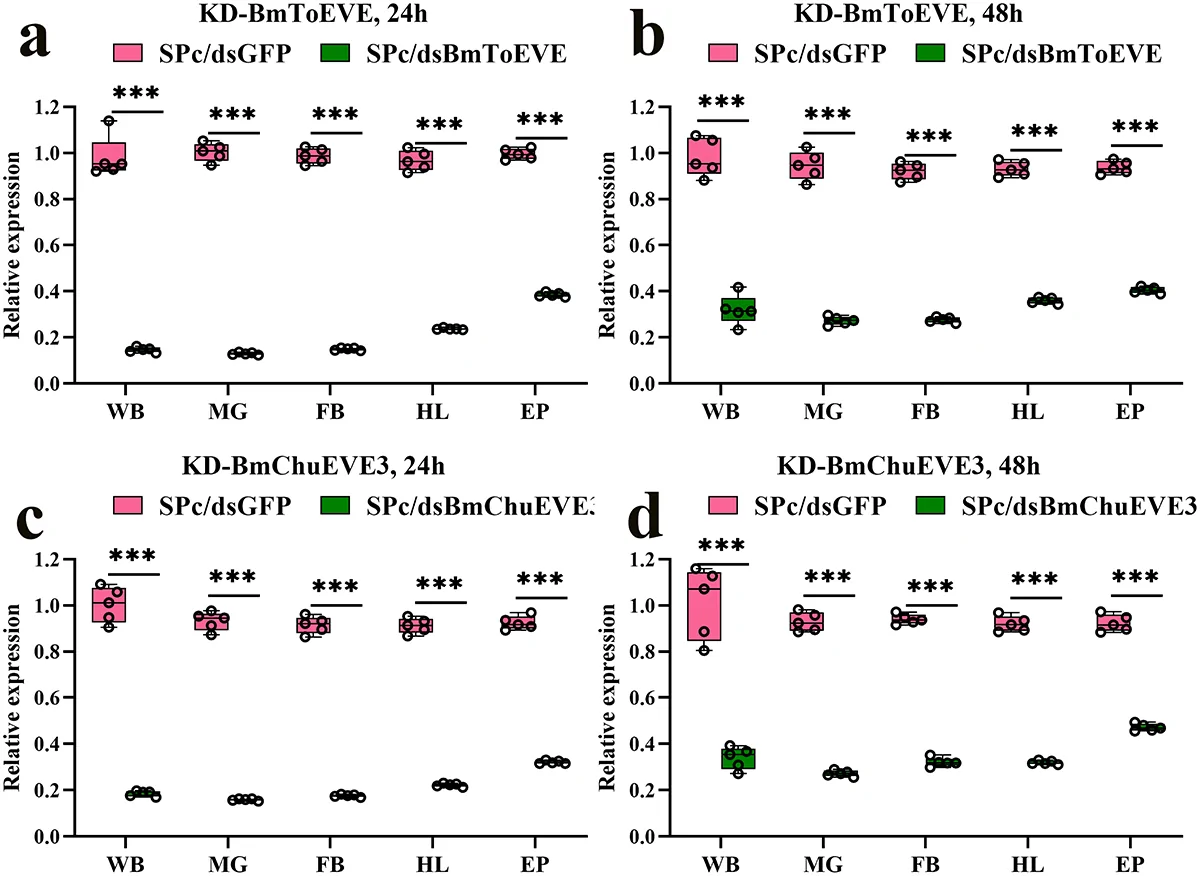
Oral SPc/dsRNA delivery efficiently silences BmToEVE and BmChuEVE3 across multiple tissues of *B. mori*. Knockdown efficiencies of BmToEVE and BmChuEVE3 were determined by qPCR after oral delivery of SPc-complexed dsRNA. (a, b) Relative expression of BmToEVE at 24 and 48 h after feeding with SPc/dsBmToEVE, respectively. (c, d) Relative expression of BmChuEVE3 at 24 and 48 h after feeding with SPc/dsBmChuEVE3, respectively. Larvae fed with SPc/dsGFP served as controls. Expression was examined in whole-body samples and in the midgut, fat body, hemolymph, and epidermis and was normalized to the corresponding SPc/dsGFP control. Individual points represent biological replicates. Statistical comparisons were performed between the SPc/dsGFP and corresponding SPc/dsRNA groups within each sample type. ***p < 0.001. WB, whole body; MG, midgut; FB, fat body; HL, hemolymph; EP, epidermis.

Given our preceding findings that BmToEVE and BmChuEVE3 exhibit markedly distinct expression peaks (Figure 2(a)) and fundamentally divergent small RNA profiles (Figure 3 and 4), we next examined their biological functions by RNAi. Oral delivery of SPc-complexed dsRNA efficiently reduced the expression of both nrEVEs. At the whole-body level, BmToEVE expression was reduced by approximately 85% at 24 h and 67% at 48 h relative to the SPc/dsGFP control (Figure 5(a,b)). BmChuEVE3 showed similarly efficient knockdown, with reductions of approximately 81% and 66% at 24 and 48 h, respectively (Figure 5(c,d)). To determine whether the feeding treatment also produced knockdown beyond the gut, we further quantified target expression in the FB, HL, and EP. Both BmToEVE and BmChuEVE3 were significantly reduced in all examined samples at 24 and 48 h after SPc/dsRNA feeding (Figure 5(a–d)). Notably, substantial knockdown was maintained in the epidermis, supporting the use of this feeding-based RNAi approach for subsequent analyses of epidermal and cuticular phenotypes.

**Figure 6.**
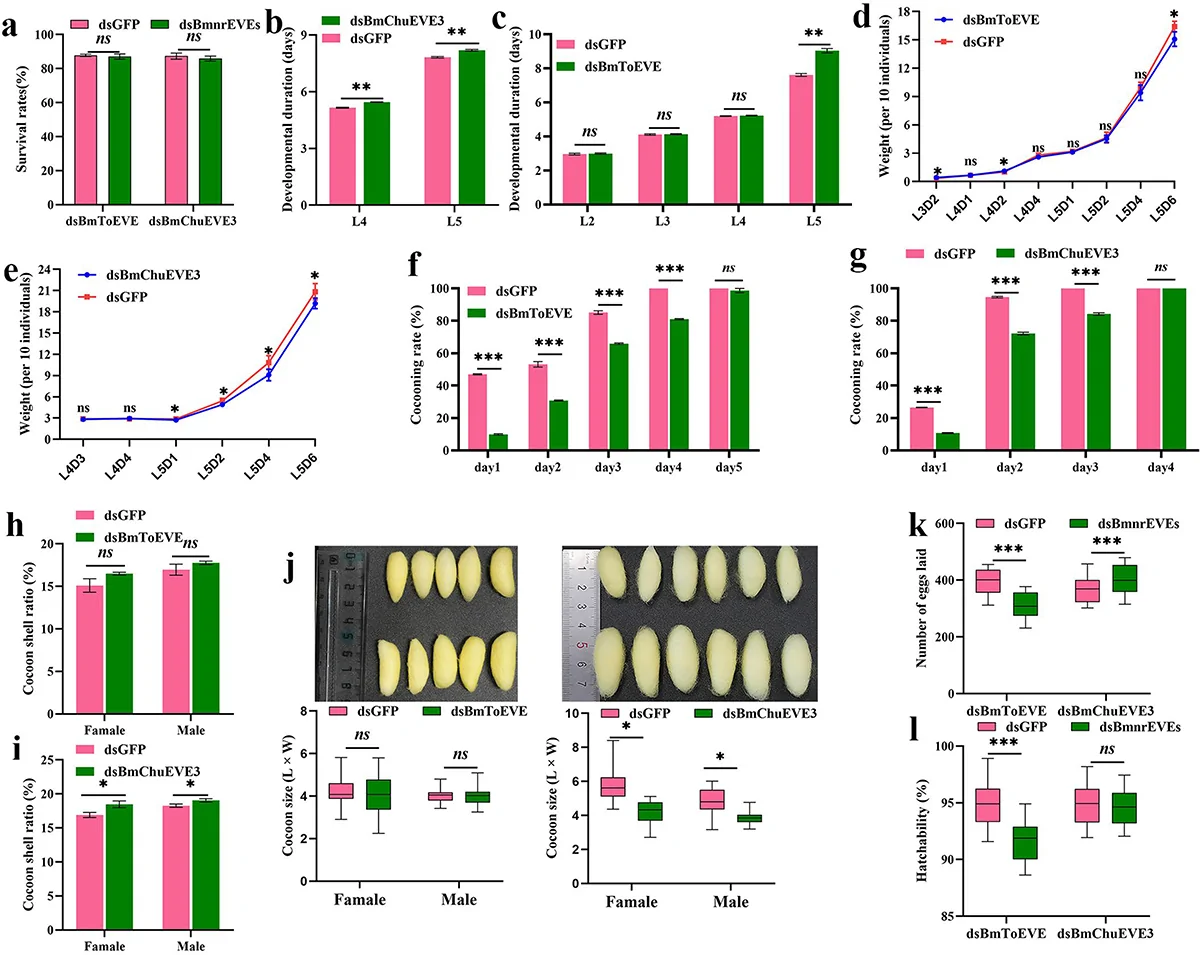
Knockdown of BmToEVE and BmChuEVE3 impairs silkworm development, growth, and reproduction. (a) Pre-cocooning larval survival rate (30 larvae per replicate). (b, c) Mean duration of each larval instar after knockdown initiation at L2D2 or L4D3 (30 larvae per replicate). (d, e) Larval body weight following knockdown initiated at (f) L2D2 or (g) L4D3 (30 larvae per replicate). (f, g) Daily cumulative cocooning rate until all larvae entered the cocooning stage (30 larvae per replicate). (h, i) Cocoon shell ratio (30 cocoons per replicate). (j) Cocoon morphology and size quantification (25 cocoons per replicate). (k) Fecundity, measured as the number of eggs laid per female (12 mated pairs per replicate). (l) Egg hatchability assessed from 12 egg masses per replicate. For all knockdown experiments, dsRNA (2 µL at 3 µg/µL) targeting BmToEVE or BmChuEVE3 was injected at L2D2 or L4D3, and injections were repeated every 48 h until pupation. Data are from three independent biological replicates and presented as mean ± SEM. Statistical significance was determined by Student’s *t*-test (*p < 0.05, **p < 0.01, ***p < 0.001, *ns*: no significance).

Systemic knockdown of BmToEVE initiated at L2D2 did not affect overall larval survival (Figure 6(a)) but induced a spectrum of specific developmental defects. BmToEVE knockdown significantly prolonged the fifth instar (Figure 6(c)) and reduced larval body weight at L3D2, L4D2, and L5D6 (Figure 6(d)). These developmental abnormalities culminated in delayed cocooning. The daily mounting rate was significantly reduced, and the average time required for cocoon formation was extended to 3.12 days, compared with 2.11 days in dsGFP-treated controls (Figure 6(f)). Despite these developmental effects, neither cocoon size nor cocoon shell ratio differed significantly between the two groups (Figure 6(h,j)). In addition to its developmental effects, BmToEVE knockdown severely impaired reproductive performance. Both fecundity and egg hatchability were significantly reduced relative to controls (Figure 6(k,l)), indicating a critical role for BmToEVE in reproductive success.

BmChuEVE3 knockdown initiated at L4D3 produced a partly overlapping developmental phenotype. Larval development was significantly prolonged, accompanied by reduced body weight during the fifth instar (Figure 6(b,e)). Mounting and cocoon formation were likewise delayed, with an average cocooning duration of 2.32 days compared with 1.79 days in control larvae (Figure 6(g)). In contrast to BmToEVE knockdown, however, suppression of BmChuEVE3 significantly altered cocoon traits. Knockdown larvae produced smaller cocoons but exhibited a higher cocoon shell ratio than controls (Figure 6(i,j)). The reproductive phenotype also differed markedly between the two nrEVEs. BmChuEVE3 knockdown significantly increased egg production, whereas egg hatchability remained unchanged (Figure 6(k,l)).

### BmToEVE and BmChuEVE3 engage distinct host protein interaction networks

**Figure 7.**
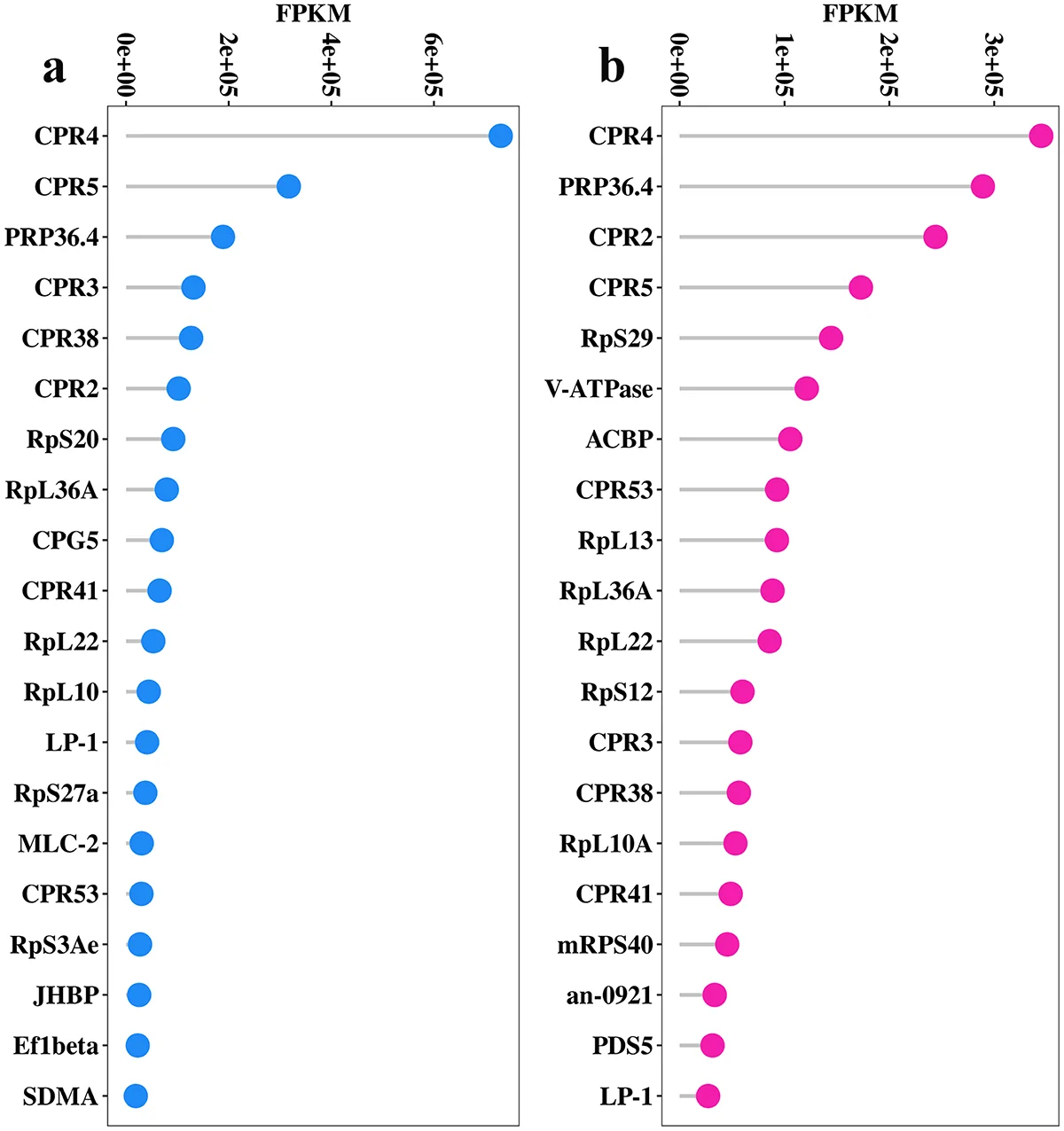
Top candidate host proteins interacting with BmToEVE and BmChuEVE3 identified by Y2H screening. the top 20 candidate interacting proteins based on FPKM are shown for (a) BmToEVE and (b) BmChuEVE3. See Supplementary Table 5 for the complete list of protein names, corresponding abbreviations, and FPKM values.

To identify host proteins potentially associated with BmToEVE and BmChuEVE3, we performed independent Y2H screens using each BmnrEVE-derived protein as bait against a *B. mori* P50 cDNA library. Positive yeast colonies were sequenced to identify the corresponding prey proteins. The BmToEVE screen recovered multiple cuticle-associated proteins, which represented a prominent class among the candidate interactors (Figure 7(a)). Frequently recovered candidates included cuticular protein RR-1 motif 4 precursor (CPR4), cuticular protein RR-1 motif 5 precursor (CPR5), and proline-rich protein 36.4 (PRP36.4). Additional members of the CPR family, including CPR2, CPR3, CPR38, and CPR41, were also identified. Other candidates included ribosomal proteins such as L22 and L36A, as well as proteins with metabolic annotations, including lipase-1 isoform X1 (Figure 7(a)). The BmChuEVE3 screen similarly recovered several cuticle-associated proteins, including CPR4 and CPR5 (Figure 7(b)). However, its candidate profile also contained proteins that were less prominent or absent from the BmToEVE screen. These included V-type proton ATPase subunit e 2 (V-ATPase), acyl-CoA-binding protein (ACBP), small ribosomal subunit protein uS14, and ribosomal protein L13.

Comparison of the two screens therefore revealed both shared and bait-specific candidate proteins. Several cuticular proteins were recovered with both BmToEVE and BmChuEVE3, whereas additional ribosomal and metabolic proteins differed between the two screens. Although the physiological relevance of these candidate associations remains to be established, these findings provide a basis for subsequent interaction and functional validation.

### BmToEVE and BmChuEVE3 knockdown drives divergent of cuticle, chorion and silk pathways

**Figure 8.**
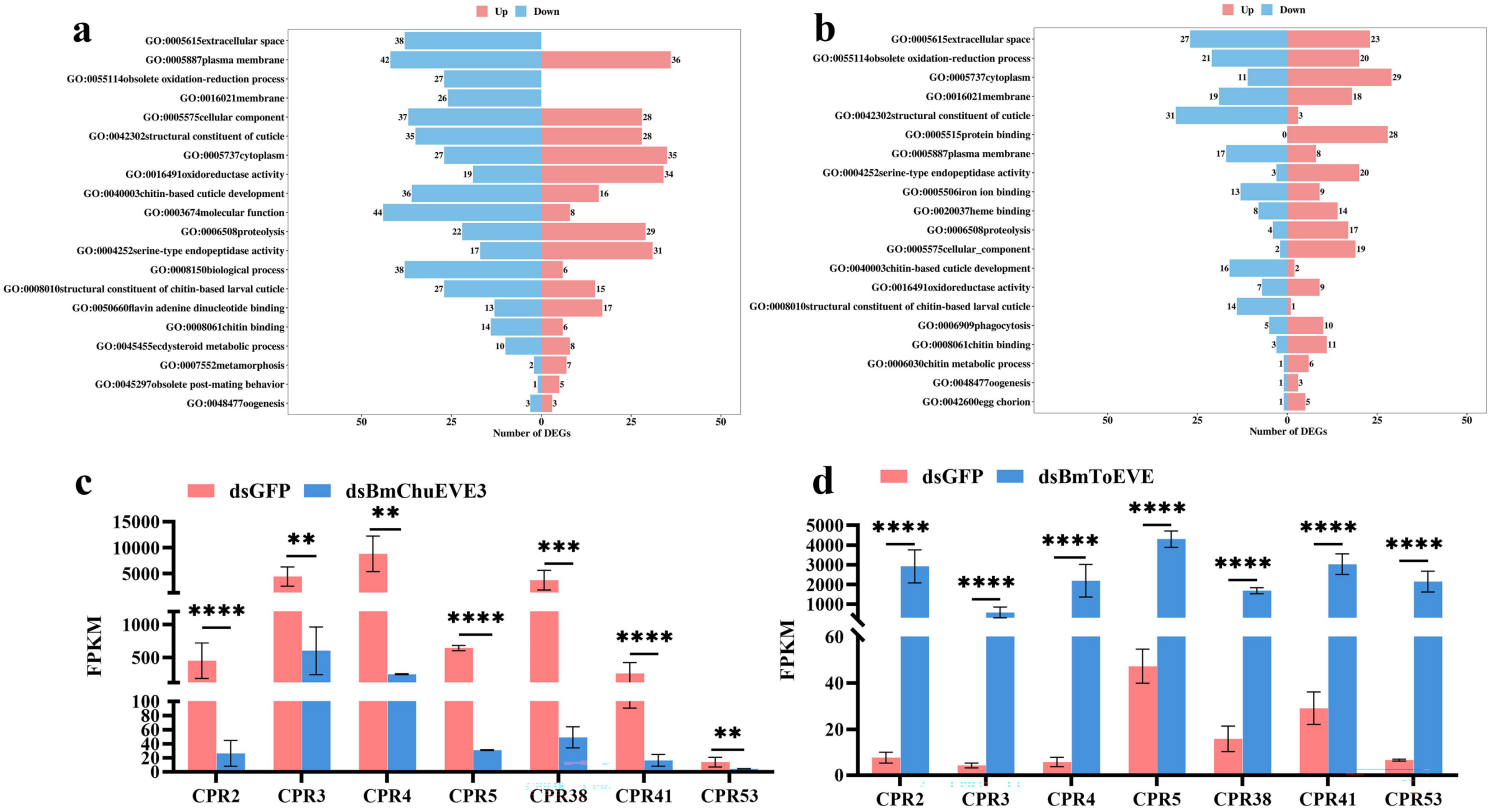
Transcriptomic responses and shared cuticular protein regulation following BmToEVE or BmChuEVE3 knockdown. (a, b) GO enrichment analysis of DEGs identified by RNA-seq after BmToEVE knockdown (L3D1) (a) and BmChuEVE3 knockdown (L5D1) (b) in silkworms. Top enriched GO terms are shown, with key terms related to cuticle development, oogenesis, and metabolism highlighted. (c, d) FPKM values of seven cuticular proteins in the control group (dsGFP) and knockdown groups (dsBmchueve3 or dsBmtoeve). These proteins were previously identified as common interactors of BmChuEVE3 and BmToEVE through Y2H screening. These proteins exhibit opposing expression patterns in response to the two nrEVE knockdowns. Statistical significance is indicated as *p < 0.05, **p < 0.01, ***p < 0.001, ****p < 0.0001.

To characterize the molecular pathways responsive to BmnrEVEs perturbation, RNA-seq was performed at developmental stages near the respective expression peaks of BmToEVE and BmChuEVE3. BmToEVE-knockdown larvae were analyzed at L3D1, whereas BmChuEVE3-knockdown larvae were analyzed at L5D1. A comparison between BmToEVE-knockdown and control samples identified 1288 DEGs, including 643 upregulated and 645 downregulated genes. GO enrichment analysis revealed a prominent representation of cuticle-related terms, such as “structural constituent of cuticle,” “structural constituent of chitin-based larval cuticle,” and “chitin-based cuticle development” (Figure 8(a)). Most genes assigned to these categories were downregulated following BmToEVE knockdown, indicating a broad perturbation of cuticle-associated transcription during larval development (Figure 8(a)). Additional DEGs were enriched in biological processes related to oogenesis, metamorphosis, and ecdysteroid metabolism (Figure 8(a)). These transcriptional changes were consistent with the developmental and reproductive phenotypes observed after BmToEVE knockdown.

BmChuEVE3 knockdown produced a distinct transcriptional response, with 603 DEGs comprising 350 upregulated and 253 downregulated genes. Among the upregulated genes, GO terms associated with oogenesis and chorion formation were significantly enriched (Figure 8(b)), indicating altered expression of reproduction-associated genes during the early fifth instar. This transcriptional pattern paralleled the increased egg production subsequently observed in BmChuEVE3-knockdown animals.

In addition, two major fibroin components, fibroin heavy chain (Fib-H; log_2_FC = 2.74, *p* = 0.029) and fibrohexamerin (P25; log_2_FC = 2.40, *p* = 0.003), were significantly upregulated. Their increased expression was consistent with the higher cocoon shell ratio observed following BmChuEVE3 knockdown. In contrast, downregulated genes were prominently enriched in cuticle-related categories (Figure 8(b)), revealing concurrent disruption of cuticle-associated transcription.

Integration of the transcriptomic data with the Y2H screening results further revealed differential responses among cuticle-associated candidates. Seven cuticular genes identified in the Y2H screens displayed opposite transcriptional responses to the two knockdowns. Their expression was reduced after BmChuEVE3 knockdown but elevated after BmToEVE knockdown (Figure 8(c,d)). Thus, although cuticle-associated genes were broadly affected by both treatments, individual candidates displayed nrEVE-specific transcriptional responses. Together, these results show that BmToEVE and BmChuEVE3 knockdown elicits distinct transcriptional changes in developmental, reproductive, and silk-related gene sets. These molecular responses broadly parallel their different phenotypic effects. However, the functional relationships between the affected pathways, the Y2H-derived candidate proteins, and the corresponding developmental and reproductive phenotypes remain to be established.

### BmnrEVEs exhibit virus-specific antiviral activities in *B.* *mori*

**Figure 9.**
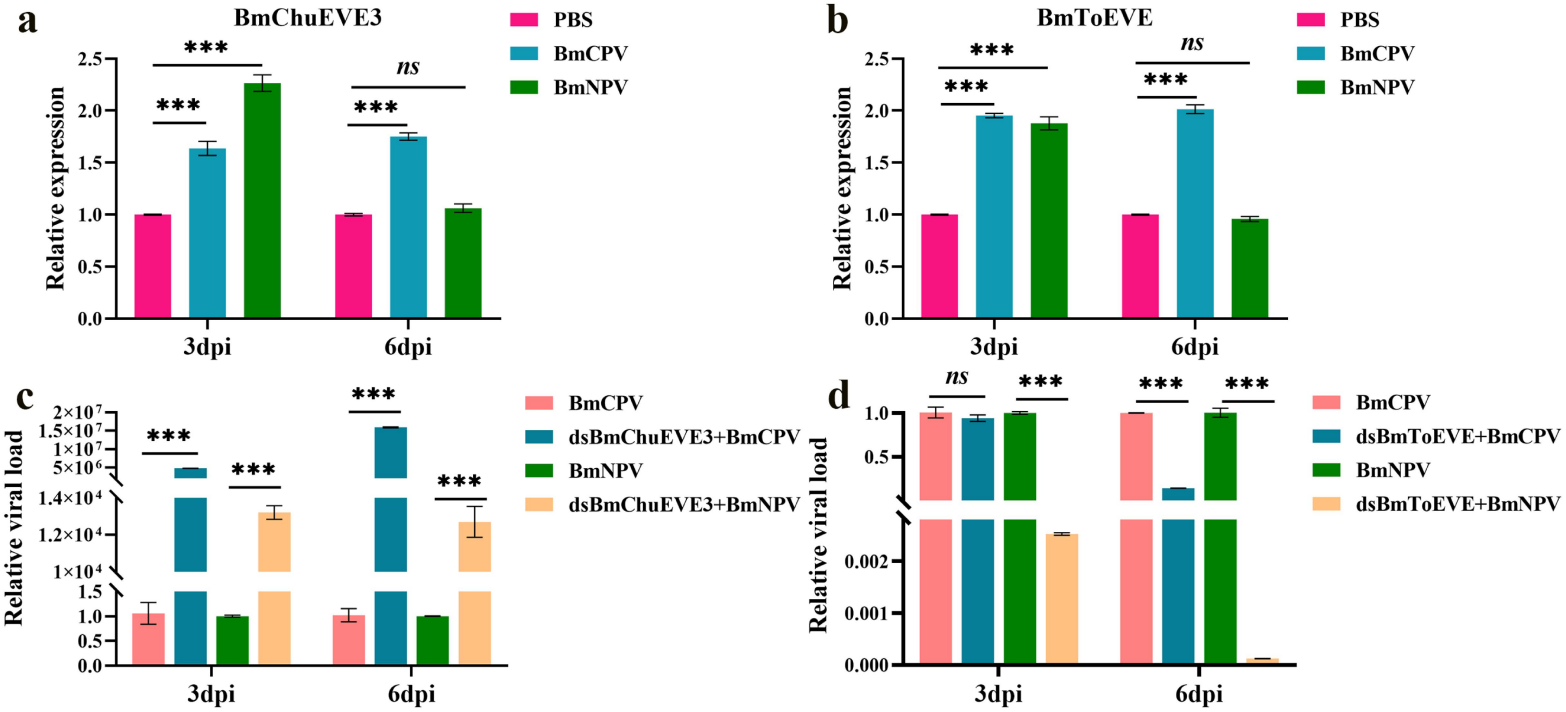
Virus-dependent expression and differential effects of nrEVEs on viral replication in silkworm larvae. (a, b) Relative mRNA levels of BmChuEVE3 (a) and BmToEVE (b) in larvae at 3 and 6 dpi. Fifth-instar day-1 (L5D1) larvae were orally inoculated with BmNPV or BmCPV, and mock-infected controls were fed PBS-treated leaves. (c, d) BmChuEVE3 and BmToEVE knockdown differentially affects viral replication. Viral genomic levels of BmNPV and BmCPV were measured by qPCR at 3 and 6 dpi following knockdown of BmChuEVE3 (c) or BmToEVE (d). Larvae were fed mulberry leaves containing gene-specific or GFP-control dsRna/spc complexes at L4D3 and L5D2 and orally inoculated with virus at L5D1. Data are presented as mean ± SEM from four biological replicates, each consisting of 15 larvae. Statistical significance was assessed against the corresponding dsGFP control using a two-tailed unpaired Student’s *t*-test (***, *p* < 0.001; ns, not significant).

To investigate the role of BmnrEVEs in silkworm antiviral immunity, fifth-instar day-1 larvae were infected with two prevalent silkworm viruses, BmNPV and BmCPV. The transcript levels of two nrEVEs, BmToEVE and BmChuEVE3, were quantified in whole larvae at 3 and 6 dpi. At 3 dpi, the mRNA levels of both BmToEVE and BmChuEVE3 were significantly up-regulated in virus-infected larvae compared to the mock-infected controls (Figure 9(a,b)). Notably, by 6 dpi, this up-regulation was sustained only in BmCPV-infected larvae. In BmNPV-infected larvae, the expression of both nrEVEs returned to levels comparable with the control group (Figure 9(a,b)).

To assess whether knockdown of these two nrEVEs affects viral replication, viral loads were compared between dsRNA-treated and control larvae. The results revealed strikingly opposing roles for the two nrEVEs. Knockdown of BmChuEVE3 significantly enhanced the replication of both viruses (Figure 9(c)). Compared with the control, BmCPV relative viral loads increased by 5.09 × 10^6^-fold at 3 dpi and 1.62 × 10^7^-fold at 6 dpi, while BmNPV loads increased by 1.32 × 10^4^-fold and 1.27 × 10^4^-fold at the respective time points (Figure 9(c)). The consistent elevation of viral loads across both viruses and time points indicates that BmChuEVE3 normally functions to restrict viral replication during infection. Conversely, knockdown of BmToEVE markedly suppressed BmNPV replication (Figure 9(d)). At both 3 dpi and 6 dpi, BmNPV viral loads were decreased by approximately 396- to 8,203-fold (Figure 9(d)), demonstrating a strong inhibitory effect of BmToEVE knockdown on BmNPV accumulation. However, knockdown of BmToEVE did not affect BmCPV loads at 3 dpi and caused only a modest reduction at 6 dpi (approximately 7-fold, Figure 9(d)). This indicates that its antiviral role against BmCPV is limited and manifests mainly at later infection stages. Collectively, these results demonstrate that BmChuEVE3 functions as a broad antiviral factor restricting both BmCPV and BmNPV replication, whereas BmToEVE exhibits a virus-specific effect, predominantly influencing BmNPV replication. The distinct outcomes observed upon knockdown of the two nrEVEs highlight functional divergence in their antiviral roles during silkworm viral infection.

### Distinct transcriptional programs mediated by BmChuEVE3 and BmToEVE in response to BmCPV and BmNPV

**Figure 10.**
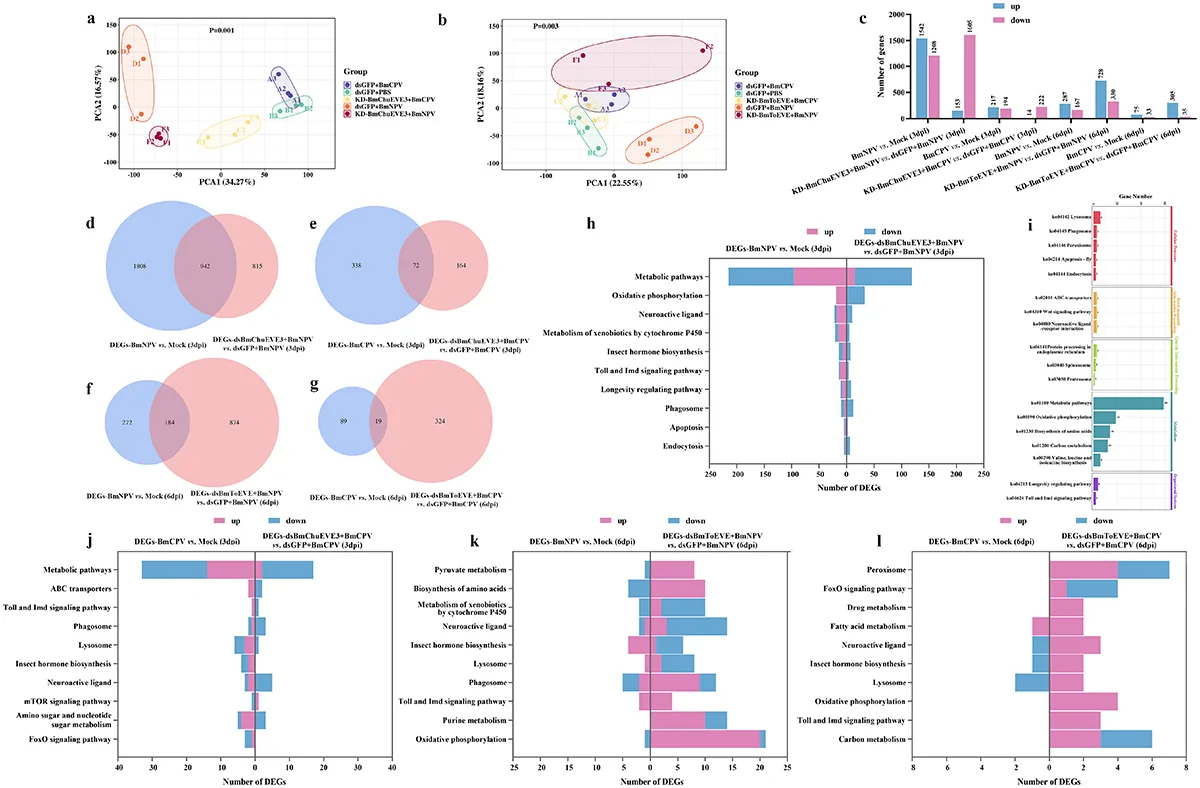
Opposing roles of BmChuEVE3 and BmToEVE in modulating host responses to BmNPV and BmCPV infections. (a, b) Principal component analysis (PCA) of transcriptomic profiles across experimental groups (BmNpv/bmcpv infection, gene knockdown ± infection, mock control). Tight within-group clustering and clear between-group separation confirm transcriptomic consistency and treatment-specific divergence. (c) Number of upregulated and downregulated DEGs in each treatment group. (d–g) Venn diagrams showing overlapping DEGs between infection-only and gene-knockdown + infection groups (for BmNPV/BmCPV infections, respectively). (h–l) KEGG pathway enrichment of DEGs: (h) BmNPV infection vs BmChuEVE3 knockdown + BmNPV; (i) the pathway enrichment for DEGs reversed by BmChuEVE3 knockdown; (j) BmCPV infection vs BmChuEVE3 knockdown + BmCPV; (k) BmNPV infection vs BmToEVE knockdown + BmNPV; (l) BmCPV infection vs BmToEVE knockdown + BmCPV. Pathways are ordered by the number of DEGs (upregulated = pink, downregulated = blue).

To elucidate the roles of BmChuEVE3 and BmToEVE in modulating the antiviral transcriptional response of the silkworm, a comparative transcriptomic analysis was conducted. Based on the viral-load profiles shown in Figure 9(c,d), samples collected at 3 dpi for BmChuEVE3 knockdown and 6 dpi for BmToEVE knockdown were selected for transcriptomic analysis. Principal component analysis (PCA) showed close clustering of biological replicates and clear separation among treatment groups (Figure 10(a,b)). These patterns confirm robust transcriptional consistency within groups and substantial transcriptomic divergence between groups, validating the dataset for subsequent analyses. We first examined BmChuEVE3-associated transcriptional changes during BmNPV infection. BmNPV infection in dsGFP-treated controls induced 2,750 DEGs, including 1,542 upregulated and 1,208 downregulated genes. BmChuEVE3 knockdown during infection resulted in 1,758 DEGs relative to infected dsGFP controls, with a strong bias toward downregulation (1,605 downregulated and 153 upregulated; Figure 10(c)). Among the 942 shared DEGs, a pronounced reversal was observed (Figure 10(d)). 797 infection-upregulated genes were suppressed upon knockdown, while 53 infection-downregulated genes became elevated. This results suggest that BmChuEVE3 knockdown substantially reverses rather than initiates a new transcriptional state.

BmNPV-responsive genes were enriched in immune-related pathways, including Toll/Imd signaling, phagosome, and endocytosis. Following BmChuEVE3 knockdown, genes associated with these pathways were predominantly downregulated (Figure 10(h)). A particularly strong effect was observed for oxidative phosphorylation, with all 33 DEGs assigned to this pathway showing reduced expression. Furthermore, genes that were oppositely regulated by BmNPV infection and BmChuEVE3 knockdown were further enriched in oxidative phosphorylation, carbon and amino acid metabolism, phagosome, and Toll/Imd signaling (Figure 10(i)). Together, these results indicate that BmChuEVE3 depletion is associated with broad suppression of metabolic and immune-related transcriptional programs during BmNPV infection. This transcriptional suppression coincided with the increased BmNPV load observed following BmChuEVE3 knockdown (Figure 9(c)).

A similar transcriptional pattern was observed during BmCPV infection. A total of 410 DEGs were detected in response to BmCPV infection in dsGFP-treated controls. By contrast, BmChuEVE3 depletion during infection was associated with 236 DEGs relative to the infected dsGFP group. These knockdown-associated DEGs showed a strong bias toward downregulation, with 222 genes downregulated and only 14 upregulated (Figure 10(c)), consistent with the pattern observed during BmNPV infection. Seventy-two DEGs were shared between the two comparisons, representing 30.5% of the BmChuEVE3 knockdown-associated DEGs (Figure 10(e)). KEGG analysis of the BmCPV-responsive genes highlighted metabolic, lysosomal, and immune-related pathways (Figure 10(j)). BmChuEVE3 knockdown shifted the transcriptome to a suppressed state. Metabolic pathway genes were significantly downregulated, and both phagocytosis and Toll/Imd pathways turned to comprehensive inhibition (Figure 10(j)). Furthermore, several signaling pathways related to development and stress response, such as insect hormone biosynthesis and FoxO signaling, were no longer engaged in the host response upon BmChuEVE3 depletion (Figure 10(j)).

In sharp contrast to BmChuEVE3, BmToEVE exhibited opposite regulatory characteristics during BmNPV infection. A total of 456 DEGs were identified between BmNPV-infected and mock-treated control larvae. BmToEVE knockdown during infection generated a larger DEG set, with upregulated genes predominating (Figure 10(c)). The overlap between the two DEG sets was limited to only 184 genes (Figure 10(f)), representing 17.2% of the knockdown group. Pathway analysis revealed three key underlying mechanisms. First, BmToEVE knockdown selectively enhanced the oxidative phosphorylation signature during BmNPV infection (Figure 10(k)), indicating a shift in mitochondrial energy metabolism. Enhanced mitochondrial bioenergetic activity appears to not only provides sufficient energy for host immune responses but may further activate immune pathways by altering mitochondrial reactive oxygen species levels (Figure 10(k)). Second, significant enrichment of the purine metabolism pathway might limit substrates for viral DNA replication by accelerating nucleotide degradation or promoting host nucleic acid synthesis (Figure 10(k)). Third, the Toll/Imd pathways and phagosome-lysosome system were synergistically activated, significantly enhancing the host’s ability to clear viruses. These findings explain the reduced BmNPV load following BmToEVE knockdown.

The proviral mechanism of BmToEVE was also evident in BmCPV infection, manifesting as potent suppression of the host response. BmCPV infection alone induced 108 DEGs, but the number of DEGs surged to 343 when BmCPV infection was combined with BmToEVE knockdown. Only 19 overlapping genes were identified between the two DEG sets, and their expression trends were completely opposite (Figure 10(g)). This indicates that BmToEVE depletion thoroughly reshapes the host transcriptional response to BmCPV, shifting toward a strong antiviral state characterized by extensive gene activation. KEGG analysis further showed that BmToEVE depletion broadly enhanced host transcriptional programs during BmNPV infection (Figure 10(l)). Upregulated DEGs were enriched in Toll and Imd signaling, carbon metabolism and oxidative phosphorylation, FoxO signaling, and cytochrome P450-associated metabolism. Together, these changes indicate coordinated enhancement of immune, metabolic, stress-response, and detoxification programs following BmToEVE depletion.

## Discussion

Non-retroviral endogenous viral elements (nrEVEs) have emerged as a pervasive but poorly understood component of animal genomes, particularly in insects [48–50]. Previous studies have shown that arthropod nrEVEs can be transcriptionally active [51], generate small RNAs [52,53], and, in some cases, acquire biological functions in their hosts [19,37]. However, whether different nrEVEs within the same host follow similar functional trajectories has remained unclear. Here, we systematically characterized nrEVEs in *B. mori* at the transcriptional, sRNAs, and functional levels. Although all identified BmnrEVEs were transcriptionally active, they differed markedly in sRNA biogenesis and functional consequences. Our findings not only establish nrEVEs as biologically active host components in Lepidoptera but also reveal their substantial functional diversity.

The predominance of negative-sense RNA virus-derived nrEVEs in *B. mori* is broadly consistent with surveys across arthropods [22,50]. Similar nrEVE diversity has now been documented in several Lepidoptera. Rhabdovirus-like EVEs are actively transcribed in *Spodoptera frugiperda* cells [54]. More recently, diverse and transcriptionally active nrEVEs have been identified in *Chilo suppressalis* and *Spodoptera* species [38,39]. Our data show that all seven BmnrEVEs are transcribed, with distinct stage- and tissue-dependent patterns. This adds to a growing body of evidence that lepidopteran nrEVEs are not uniformly transcriptionally silent. Of particular interest, several BmnrEVEs reached relatively high expression during post-ecdysial feeding periods, suggesting possible responsiveness to developmental cues.

Small-RNA profiling revealed pronounced differences in the processing of individual BmnrEVEs. All BmnrEVEs produced abundant ~20-nt RNAs from both strands. In addition, longer small RNAs were detected at multiple loci and often showed strong strand bias and nucleotide preferences characteristic of piRNAs. The piRNA-like reads also differed among BmnrEVEs. BmChuEVE1, BmChuEVE2, and BmToEVE displayed a prominent 10-nt 5' overlap between oppositely oriented small RNAs. This signature was accompanied by complementary 1 U- and 10A-associated nucleotide biases, consistent with ping-pong-associated amplification. By contrast, BmChuEVE3 and BmPhaEVE predominantly produced strand-biased, 1 U-enriched piRNA-sized RNAs without a clear 10-nt overlap. These differences are consistent with previous arthropod studies showing that EVEs commonly generate piRNAs, but differ substantially in their abundance and biogenesis [50,55]. Recent work in *Spodoptera* similarly reveals marked variation in piRNA production among individual nrEVEs [39].

The recurrent ~20-nt, bidirectional small-RNA class represents a second notable feature of BmnrEVEs. This size and strand distribution are compatible with siRNA-like processing. In *B. mori*, BmCPV infection produces abundant ~20-nt virus-derived small RNAs associated with the Dicer-2-dependent antiviral RNAi pathway [56,57]. Reverse-transcribed BmCPV-derived DNA can also serve as a template for antiviral siRNA production [58]. Notably, EVE-derived siRNAs were infrequently detected in a comparative analysis of arthropod EVEs [50]. The consistent detection of ~20-nt sRNAs from silkworm nrEVEs may therefore reflect a distinctive mode of nrEVE-derived sRNA processing. However, their assignment to the canonical siRNA pathway remains provisional. The ability of endogenous siRNAs to target cellular transcripts raises the possibility that nrEVE-derived siRNAs may also participate in host gene regulation in trans [59]. Integrating target-prediction analyses with AGO2-associated sRNA profiling and experimental validation will help determine whether such siRNA contribute to host gene regulation [60].

An important implication of our findings is that BmnrEVE function may extend beyond small-RNA biogenesis. We found that BmToEVE and BmChuEVE3 are required for normal silkworm development. This functional role is reminiscent of NlToEVE14, a totivirus-derived EVE that has been co-opted for host physiology in *N. lugens* [37]. NlToEVE14 interacts with the cuticle-associated protein NlTwd, linking this virus-derived sequence to host developmental processes. In *B. mori*, both BmToEVE and BmChuEVE3 were similarly associated with cuticular proteins and cuticle-related transcriptional responses. These observations raise the possibility that virus-derived sequences have been independently recruited for developmental regulation across different insect taxa. The distinct reproductive and cocoon phenotypes associated with the two BmnrEVEs further argue against simple functional redundancy and suggest that their physiological roles may have diverged during host co-option. However, the molecular basis of these effects remains unresolved. An additional limitation is that larval developmental traits were evaluated in mixed-sex populations, whereas the reproductive assays involved mating between males and females subjected to the same knockdown treatment. The present data therefore do not establish whether the developmental or reproductive effects of BmToEVE and BmChuEVE3 are sex dependent. This distinction may be important because male and female silkworms differ in several physiological and cocoon-related traits [61,62]. Future studies will use stable knockout and overexpression lines to examine sex-specific effects. These genetic alterations will be introduced into sex-limited strains to enable early sex discrimination during larval development. Reciprocal mating between genetically manipulated and control males and females will further distinguish maternal and paternal contributions to the reproductive phenotypes.

The effects of BmToEVE and BmChuEVE3 on viral infection reveal a distinct mode of nrEVE function. The best-characterized antiviral nrEVE systems in mosquitoes rely on sequence complementarity between EVE-derived piRNAs and cognate or closely related viral RNAs [35,46]. Such a mechanism is unlikely to fully explain our observations because BmToEVE and BmChuEVE3 are evolutionarily unrelated to the two viruses examined here. Instead, the contrasting infection phenotypes and associated transcriptional responses point to a host-centered mechanism. BmChuEVE3 depletion was accompanied by reduced expression of immune- and metabolism-related pathways together with increased viral accumulation, whereas BmToEVE depletion during BmNPV infection enhanced innate immune, phagosome-related, oxidative phosphorylation, and FoxO-associated programs and coincided with reduced viral abundance. These pathways have established roles in silkworm antiviral defense; for example, BmSTING promotes Dredd-dependent NF-κB activation, while BmFoxO enhances resistance to BmNPV [63–65]. Together, these findings suggest that BmChuEVE3 and BmToEVE may shape virus susceptibility by altering host physiological and transcriptional states. This consistent with emerging evidence that insect nrEVEs can be co-opted into host regulatory functions [37], rather than functioning exclusively as sources of sequence-matched antiviral small RNAs [35,50]. Whether these effects are mediated by nrEVE-encoded proteins, or downstream host pathways remains to be determined.

These insights significantly advance our understanding of nrEVE biology. Rather than acting solely as sources of virus-derived small RNAs, BmToEVE and BmChuEVE3 are associated with distinct effects on development, reproduction, and viral infection. However, the molecular basis of these effects remains unresolved. Furthermore, comparative analyses across silkworm populations and other lepidopteran species will also be required to clarify the evolutionary history and selective forces underlying the retention of these elements. Importantly, unraveling these mechanisms will be crucial for assessing the potential of nrEVEs as genetic targets for improving traits in economically important insects.

## Ethics and compliance

Ethical approval was not required for this study as it involved only the insect species *B. mori*. No experiments were performed on humans or vertebrate animals.

## Supplementary Material

Supplementary Table 1.xlsx

Supplementary Table 3.xlsx

Supplementary Table 5.xlsx

Supplementary Table 4.xlsx

Supplementary Table 2.xlsx

## Data Availability

The data supporting the findings of this study, including the raw data underlying the figures and statistical analyses, are available at Figshare (doi: https://doi.org/10.6084/m9.figshare.31441696) [66]. Detailed Excel files for the RNA-seq differential expression and yeast two-hybrid analyses are also provided in this repository. The RNA-seq data are available through the public database Genome Sequence Archive (GSA) under BioProject PRJCA059014, https://ngdc.cncb.ac.cn/ bioproject/browse/PRJCA059014) [67].
